# Evaluation of hybrid DIR performance using controlling structures and points of interest in MR‐guided adaptive radiotherapy for prostate cancer patients

**DOI:** 10.1002/acm2.70437

**Published:** 2026-01-14

**Authors:** Victor Malkov, Iymad R. Mansour, Vickie Kong, Winnie Li, Jennifer Dang, Parisa Sadeghi, Inmaculada Navarro, Jerusha Padayachee, Peter Chung, Jeff D. Winter, Tony Tadic

**Affiliations:** ^1^ Mayo Clinic Department of Radiation Oncology Rochester Minnesota USA; ^2^ Radiation Medicine Program Princess Margaret Cancer Centre Toronto Ontario Canada; ^3^ Department of Radiation Oncology University of Toronto Toronto Ontario Canada

**Keywords:** adaptive radiotherapy, deformable image registration, DIR, dose accumulation, MR‐linac, prostate SBRT

## Abstract

**Background:**

MR‐guided adaptive radiotherapy (ART) allows for daily plan optimization based on patient‐specific anatomy. Accumulated doses, driven by deformable image registration (DIR), of daily fractions can provide cumulative dose metrics and insights into toxicity and tumor control. In prostate ART, inter‐ and intra‐factional deformations, particularly due to bladder and rectum, pose a challenge to accurate DIR generation.

**Purpose:**

To quantify geometric and dosimetric accuracy of a proposed prostate MR‐to‐MR DIR approach to support MR‐guided ART dose accumulation.

**Methods:**

We evaluated DIR accuracy in 25 patients treated with 30 Gy in five fractions on a 1.5 T MR‐linac using an adaptive workflow. For all patients, a reference MR was used for planning, with three images collected at each fraction: adapt MR for adaptive planning, verify MR for pretreatment position verification and beam‐on for capturing anatomy during radiation delivery. We assessed three DIR approaches: intensity‐based, intensity‐based with controlling structures (CS), and intensity‐based with controlling structures and points of interest (CS + P). DIRs were performed between the reference and fraction images and within fractions (adapt‐to‐verify and adapt‐to‐beam‐on). For the evaluation, we propagated CTV, bladder, and rectum contours using the DIRs and compared each to manually delineated contours using Dice similarity coefficient, mean distance to agreement, and dose–volume metrics.

**Results:**

CS and CS + P improved geometric agreement between manual and propagated contours over intensity‐only DIR. For example, mean distance to agreement (DTA_mean_) for reference‐to‐beam‐on intensity‐only DIR was 0.131 ± 0.009 cm (CTV), 0.46 ± 0.08 cm (bladder), and 0.154 ± 0.013 cm (rectum). For the CS, the DTA_mean_ values were 0.018 ± 0.002, 0.388 ± 0.14, and 0.036 ± 0.013 cm. Finally, for CS + P, these values were 0.015 ± 0.001, 0.025 ± 0.004, and 0.021 ± 0.002 cm. Dosimetrically, comparing CS and CS + P for reference to beam‐on DIRs resulted in a change of CTV D98% from [−29 cGy, 19 cGy] to [−18 cGy, 26 cGy], bladder D5cc from [−51 cGy, 544 cGy] to [−79 cGy, 36 cGy], and rectum D1cc from [−106 cGy, 72 cGy] to [−52 cGy, 74 cGy].

**Conclusion:**

CS improved geometric and dosimetric accuracy over intensity‐only DIR, with CS + P providing further performance improvement, particularly for bladder. However, session image segmentation remains a challenge, which may be addressed with automated contouring.

## 1 INTRODUCTION

Integrated MRI linear accelerator (MRL) systems offer superb soft tissue contrast and real‐time imaging during radiation delivery for targeting monitoring and gating, as well as support for online adaptive radiation therapy (ART) to tailor each treatment to the daily internal anatomy.^1^ With a daily adaptive framework, cumulative dose delivered over the full treatment course includes inter‐fractional anatomical differences, unique daily adaptive treatment plans and internal anatomical changes between adapted plan generation and beam delivery.^2^, ^3^ Quantifying accumulated delivered dose following each fraction enables monitoring of aggregate target and OAR doses relative to the original reference plan and treatment clinical goals, offering an opportunity to make informed adaptive replanning decisions for future fractions. Moreover, accumulated dose can be leveraged to evaluate and optimize adaptive strategies,^4^ as well as provide a more meaningful correlate between dose volume metrics and patient outcomes.^5^, ^6^ Using deformable image registration (DIR), it is possible to map dose to a target image set with the associated deformed vector field (DVF) enabling dose summation that can be leveraged for calculation of accumulated delivered dose on the MRL.^7^, ^8^, ^9^, ^10^ However, dose accumulation accuracy is directly related to DIR performance; as such, dose accumulation tools require careful application‐specific validation prior to clinical use.

Validation and quantification of uncertainties in the DIR used to deform dose for accumulation is a critical step required prior to clinical dose accumulation implementation in MR‐guided ART. The AAPM Task Group 132 and more recent reports describe quantitative and qualitative tools required for establishing the accuracy of deformable image registration.^10^, ^11^ Validation requires expert visual review as well as geometric and dosimetric considerations. Large anatomical changes or gain/loss of image information between the target and moving images are particularly challenging for DIR performance. Both scenarios exist in the inter‐ and intra‐fraction bladder and rectum changes within prostate MRL adaptive workflows necessitating novel DIR solutions.^12^ Recent work compared various DIR approaches currently employed at different centers for multiple treatment sites for the purpose of inter‐fraction dose accumulation.^13^ This cited study demonstrated generally high between‐institution agreement with bladder dose–volume differences being the greatest for the prostate cases.

In this work, we proposed a unique solution to account for large deformations for both inter‐ and intra‐fraction prostate MRL DIR using a hybrid image‐intensity approach based on controlling structures and points. We performed a geometric and dosimetric evaluation of this enhanced approach, including comparison with conventional intensity‐only or hybrid intensity and controlling structure DIR approaches. We establish this DIR approach for subsequent dose accumulation work in this cohort independent from this current work.

## 2 MATERIALS AND METHODS

### 2.1 Patients

We investigated DIR accuracy in a cohort of 25 patients selected continuously, who were enrolled in a prospective clinical trial investigating MR‐guided prostate brachytherapy and external beam radiation therapy (ClinicalTrials.gov Identifier: NCT00913939). A description of the patient demographics (Table S1) and inclusion criteria is provided in the Supporting Information.

The study was approved by our institution's research ethics board and all patients provided informed written consent. Patients received 30 Gy in five fractions treated on the MR‐Linac with consecutive 15 Gy in 1 high‐dose brachytherapy boost to intraprostatic gross tumor volumes. Both bladder and bowel preparation were required for all patients, including asking patients to have a bowel movement prior to simulation and each treatment fraction as well as drinking 300 mL of water at least 15 min prior to treatment.

### 2.2 MR‐linac treatment

We performed reference and online treatment planning using Monaco 5.40.01 (Elekta, Stockholm, Sweden) with a 9‐beam IMRT beam geometry designed to meet our institutional dose constraints (Table S2). Monte Carlo dose was calculated on a 3 mm grid incorporating the 1.5 T magnetic field with 1% statistical uncertainty. At each adaptive fraction, we acquired an MR (MR_adapt_) and performed a manual rigid registration with the reference MR based on a soft tissue match of the prostate. A radiation oncologist or fellow performed any required manual contour edits based on rigid or deformably propagated contours, focusing on the region within 2 cm of the planning target volume. Next, we generated an adapted treatment plan based on small real‐time adjustments of the starting objectives using the newly contoured structures. For dose computation, bulk densities were assigned to the external, femurs, and PTV using mean electron densities extracted from a CT image collected on the same day as the MR simulation. As part of our standard workflow, prior to treatment delivery, we acquired and reviewed a verification MR (MR_verify_) collected during plan quality assurance, followed by a 6‐min beam‐on MR (MR_beam‐on_) during beam delivery for potential offline evaluation. Unit staff will monitor the patient for gross motion. In cases in which we suspect changes since the adapted plan was generated, we will use 3‐plane CINE MR immediately prior to beam‐on for verification. Once the beam has started, we will continue delivery. Table S3 provides the imaging parameters for the MR sequences.

### 2.3 Fractional dose computation and manual contouring

For each fraction, we recomputed the fractional adapted treatment plan on each of the MR_verify_ and MR_beam‐on_ images using the adapt‐to‐shape with original segments function in offline Monaco. For dose computation, each organ‐at‐risk (OAR) maintained the density assignment determined using the reference CT image. We then imported the reference plan dose, reference MR, fractional doses and MR images (MR_adapt_, MR_verify_, and MR_beam‐on_) into RayStation 10B (RaySearch, Stockholm, Sweden). In total, we analyzed 25 reference and 375 fractional MR images of structures and doses.

For offline contouring, a radiation‐oncologist‐trained adaptive radiation therapist reviewed and edited clinical target volumes (CTV) (prostate), bladder, and rectum on the MR_reference_ and MR_adapt_, and generated manual contours on the MR_verify_ and MR_beam‐on_ in RayStation following the ESTRO ACROP recommendations.^14^ At our center, RTTs have been trained to contour prostate cases for adaptive treatment following a phased training approach.^15^ Training was provided specifically for the contouring of the targets and OARs on the verification and beam‐on scans for this study. Resulting contours were inspected and modified as required by an independent RO fellow and confirmed by a medical physicist, who flagged outliers and prompted additional contour edits and review process iterations.

### 2.4 Controlling point generation

**FIGURE 1:**
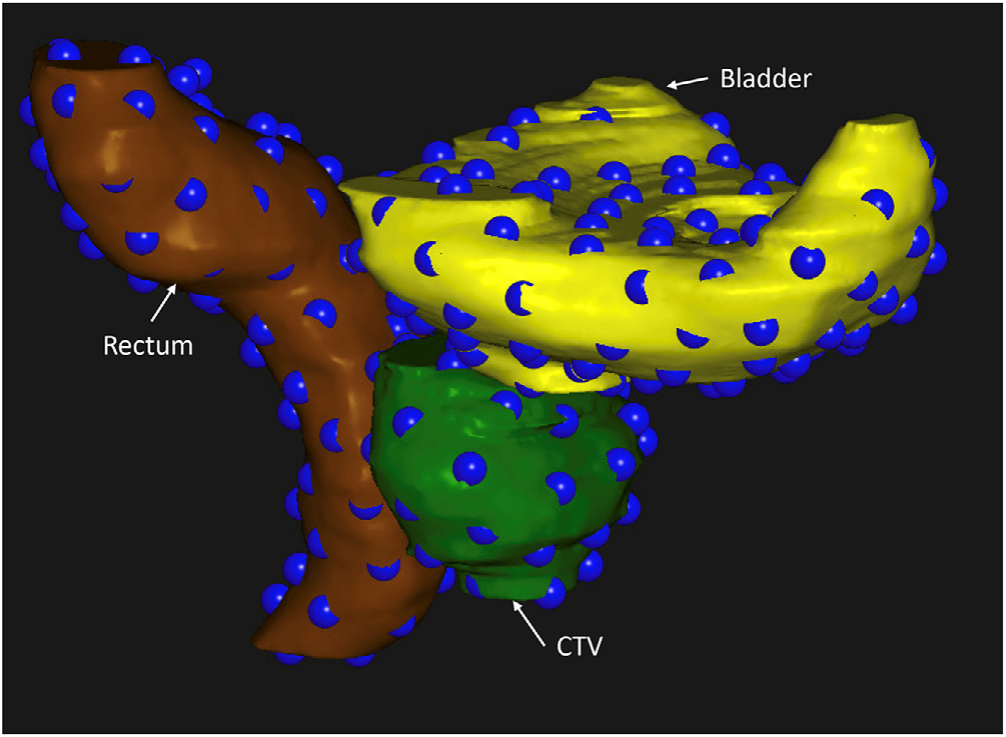
3D visualization of controlling points (blue spheres) generated on the surface of the bladder (yellow), clinical target volume/prostate (green), and rectum (brown) for a representative patient.

An in‐house developed script automatically generated anchor points on the surface of the bladder, rectum, and CTV structures (Figure 1) to be used as controlling points in the DIR generation. These points are meant to represent corresponding anatomical locations on the organ surfaces between the MR images. To determine these point locations, we generated mesh representations of the manually contoured structures on the reference and fractional MR images using the RayStation *create controlling ROI* function (mesh detail = 0.4). This function generates a 3D tetrahedral mesh on each image that is a smooth approximation to the manual contours. Following an approach similar to the MORFEUS biomechanical DIR algorithm,^16^ each mesh is based on a common topology using the same number of vertices and indexing of edges, ensuring a direct vertex‐to‐vertex mapping. This vertex‐to‐vertex mapping is initialized with an orthogonal surface projection between image pairs and is not driven by the identification of distinct anatomical features in the images. This approach yields a plausible ROI surface registration; however, vertex pairs between images are not guaranteed to represent identical anatomical points on the ROI surface.

The set of controlling POIs for DIR generation was selected as a subset of the mesh vertices on the reference MR. Starting from a random mesh vertex, subsequent vertices were selected ensuring a minimum separation of 10 mm as calculated using the dijkstra_path_length function from the NetworkX Python library.^17^ The selected mesh indices were then used to identify the corresponding coordinates for the controlling POIs on the fractional images. Due to the random initialization and subsequent distance‐driven selection of mesh vertices for POI creation, the locations of the resultant controlling POIs coincide with a random subset of the vertices and are not related to any particular anatomical landmarks or features of the ROI. The minimum separation of 10 mm was empirically chosen, as it yielded good sampling of ROI shape variations, based on a visual qualitative review of the POI arrangements on a subset of 10 patients. This sampling density resulted in POIs placed near the most distal aspects of each ROI and in regions of high surface curvature.

### 2.5 Deformable image registration

**FIGURE 2:**
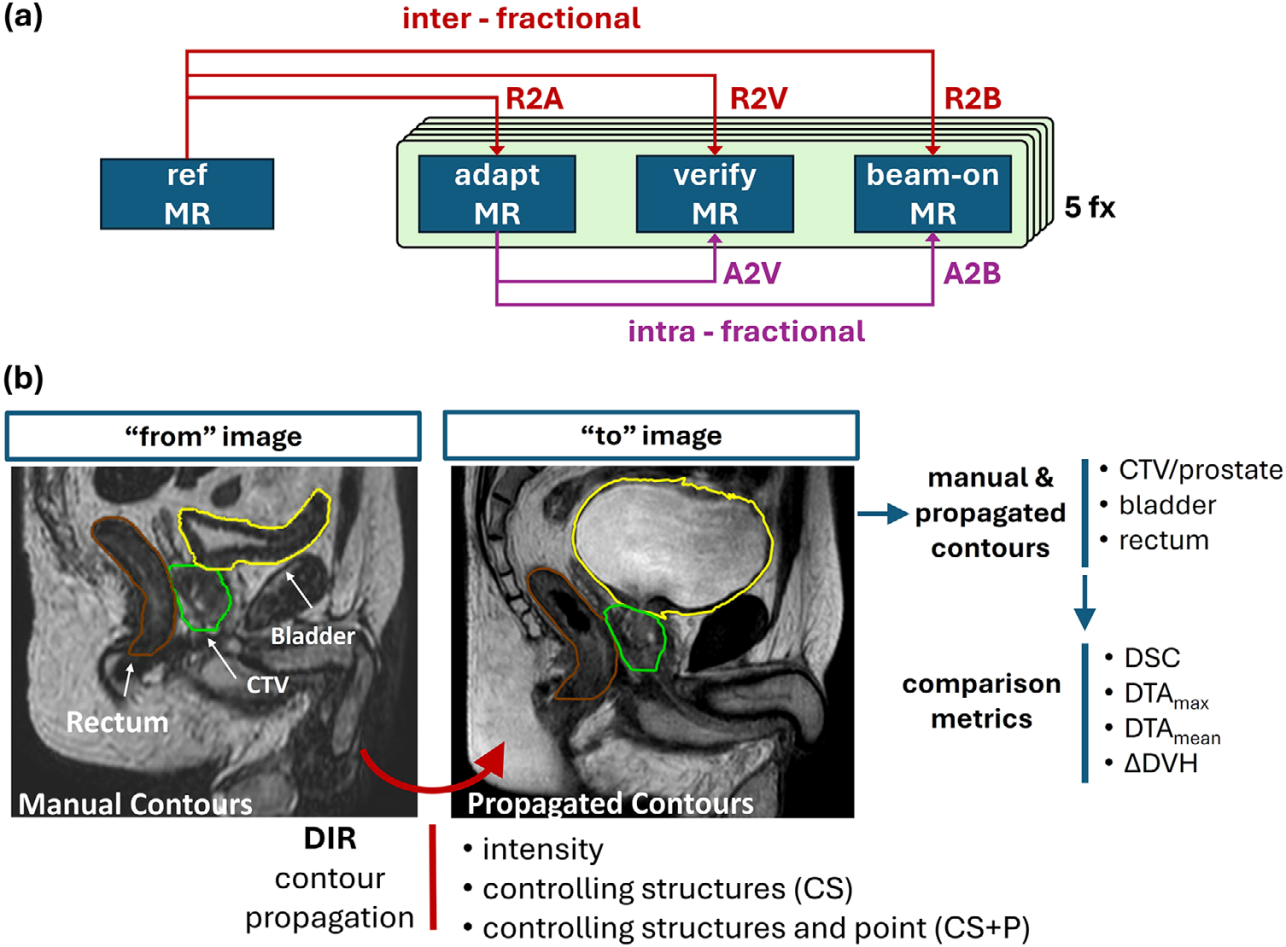
(a) Summary of image pairs (inter‐ or intra‐fractional) used in DIR generation and evaluation and (b) overview of the DIR strategies used for propagating contours between image pairs and the comparison metrics used for DIR accuracy evaluation. Three DIR strategies (intensity‐only, CS, and CS + P) are used to deform the “from” image CTV, bladder, and rectum to the “to” image. DIR evaluation is performed using geometry and DVH metrics comparing the propagated contours to the manual contours on the “to” image. CS, controlling structure; CS + P, controlling structure with points; DIR, deformable image registration.

We generate DIRs in RayStation using the hybrid intensity‐ and structure‐based algorithm called ANACONDA.^18^ Our analysis included interfraction DIRs, between the reference MR and each of the three fractional MR images, and intrafraction DIRs, between the daily planning MR (MR_adapt_) and verification (MR_verify_) and beam‐on (MR_beam‐on_) images. We use the shorthand nomenclature: R2A to represent reference to adapt MR DIRs and similarly, R2V (reference to verify), R2B (reference to beam‐on), A2V (adapt to verify), and A2B (adapt to beam‐on). Figure 2a summarizes the DIR generation and nomenclature. We followed a stepwise study design to assess potential improvement in DIR accuracy using either: 1) Image‐intensity only, 2) image‐intensity with controlling structures (CS), and 3) image‐intensity with controlling structures and points (CS + P). We utilized the manual CTV, bladder, and rectum contours as the controlling structures. Controlling points were defined for each controlling structure using the mesh representations via the method described above. Overall, for each image pair and DIR strategy 125 DIRs were performed (25 patients × 5 fractions).

### 2.6 DIR analysis

To assess DIR accuracy, we applied the DIR strategies (intensity, CS, or CS + P) to deformably propagate the bladder, rectum, and CTV manually delineated contours between the “from” and “to” images. We then performed a geometric comparison of the propagated contours with the corresponding manual contours on the “to” image by computing the volume Dice Similarity Coefficient (DSC), mean distance to agreement (DTA_mean_), and the max distance to agreement (DTA_max_) using scripting methods in RayStation.

To further assess dosimetric DIR accuracy, we extracted dose volume histograms (DVHs) for each of the propagated and manual contours using the recalculated dose distributions on each image (no dose propagation is performed). We computed the difference of these relative volume DVHs, ΔDVH, to provide a dose‐informed evaluation of the DIR accuracy. In this way, the ΔDVH represents the dosimetric error arising from geometric misalignment of the propagated and manual contours, ultimately due to a combination of manual contouring and DIR uncertainties. The ΔDVHs, per fraction dose differences, were linearly scaled by a factor of five to reflect the full five‐fraction treatment course to allow for comparison with the reference plan clinical goals. We also report a clinically relevant set of specific DVH metrics from the ΔDVH for each image pair and DIR approach: CTV D98%, bladder D5cc, and rectum D1cc. The DIR strategies and evaluation metrics are summarized in Figure 2b.

## 3 RESULTS

The results below focus on the A2B and R2B DIR, as these would be of greatest utility in the context of interfraction and intrafraction dose accumulation. Detailed results for the other DIRs are presented in the Supporting Information.

### 3.1 Intra‐fractional

**FIGURE 3:**
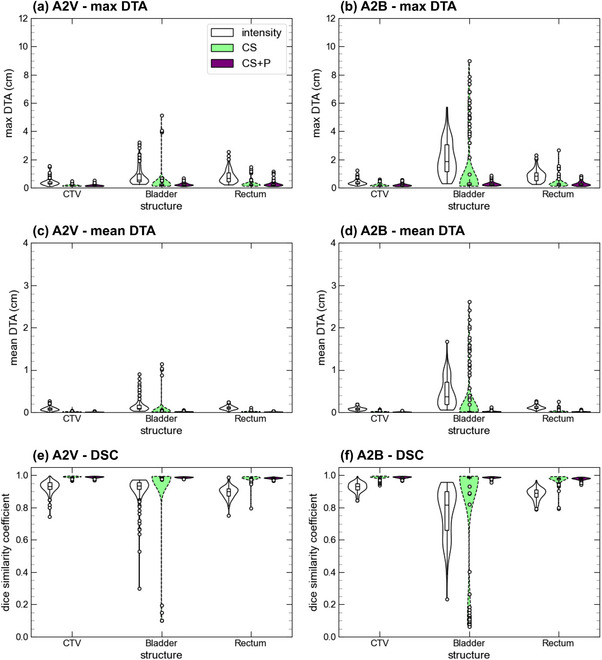
Intra‐fractional geometry metrics comparing deformed CTV, bladder, and rectum from the MR_adapt_ to the MR_verify_ (A2V) and MR_beam‐on_ (A2B) for the three DIR strategies (intensity‐only, CS, CS + P). CS, controlling structure; CS + P, controlling structure with points; DIR, deformable image registration.

**FIGURE 4:**
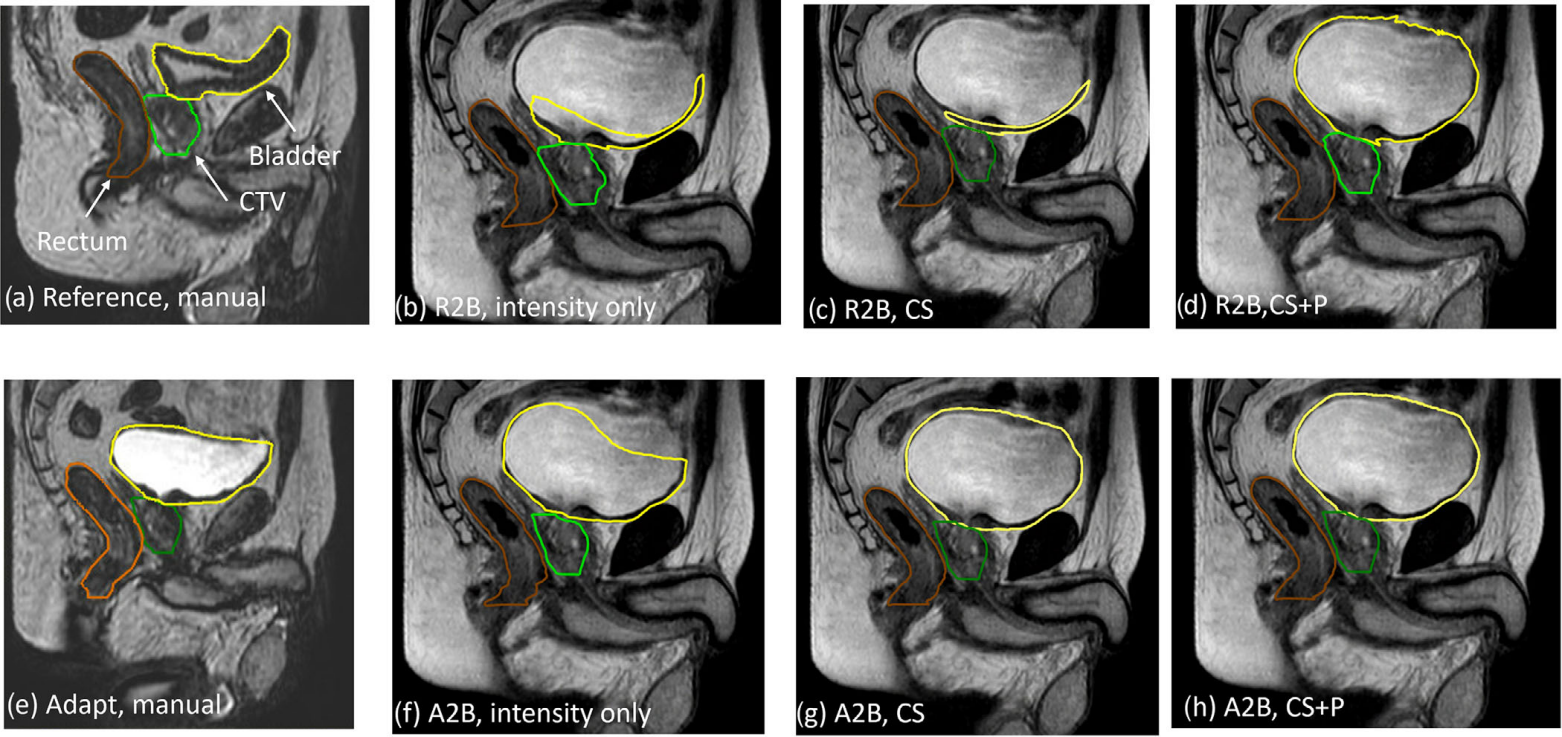
Comparison of different contour propagation methods to the beam‐on image dataset. For both the reference (a) and adapt (e) images, contours are manually created. Contours are propagated from the reference to beam‐on images (b–d) or adapt to beam‐on images (f–h) images. Propagation based on either intensity only (b and f), controlling structures (c and g), or controlling structures and points (d and h) DIR approaches are presented. DIR, deformable image registration.

In Figure 3, we provide the DTA_max_, DTA_mean_, and the DSC plots for the A2V and A2B image pairs for the intensity, CS, and CS + P DIR strategies. Table 1 provides the mean for each metric and image pair. The CS strategy produces an improvement of all metrics for the CTV and the rectum relative to the intensity‐only approach. For example, for the A2B pair, DTA_mean_ changed from 0.085 ± 0.007 cm to 0.015 ± 0.002 cm for the CTV and from 0.118 ± 0.008 cm to 0.024 ± 0.006 cm for rectum. Generally, the CS + P DIR performance did not substantially differ from the CS approach for structures with minimal geometric variations; however, measurable improvements existed for structures exhibiting large geometric variations. For example, CS + P improved the DIR performance for bladder with DTA_mean_ for A2B reducing from 0.47 ± 0.07 cm to 0.27 ± 0.12 cm between intensity and CS DIRs, with only a small subset of fractions performing worse than the intensity‐only DIR approach. This is most pronounced in the A2B comparisons and is attributed to bladder filling over the course of the adaptive fraction and is presented in further detail in the inter‐fraction results. In Figure 4, we provide the manual contours on the moving image and propagated contours using all three DIR strategies for a patient with a poor bladder deformation using the CS method. We observed that the reference image had a near‐empty bladder and resulted in poor propagation where the entire bladder is directed toward the inferior bladder surface. The CS + P strategy corrected these deviations, as seen in the improved geometry metrics for the bladder (DTA_mean_ reducing to 0.022 ± 0.003 cm for the A2B mapping).

**TABLE 1:** Intra‐fractional mean geometry metrics for the A2V and A2B image pairs for all three DIR strategies and contours.

Image pair	DIR	OAR	DTA_max_ (cm)	DTA_mean_ (cm)	DSC
A2V	Intensity	CTV	0.40 (0.05)	0.083 (0.009)	0.929 (0.007)
		Bladder	0.85 (0.13)	0.15 (0.03)	0.910 (0.018)
		Rectum	0.81 (0.09)	0.110 (0.009)	0.896 (0.007)
	CS	CTV	0.153 (0.012)	0.010 (0.001)	0.989 (0.001)
		Bladder	0.36 (0.15)	0.05 (0.04)	0.96 (0.03)
		Rectum	0.28 (0.5)	0.015 (0.002)	0.982 (0.004)
	CS + P	CTV	0.165 (0.014)	0.010 (0.001)	0.989 (0.001)
		Bladder	0.239 (0.020)	0.018 (0.001)	0.987 (0.001)
		Rectum	0.26 (0.03)	0.014 (0.001)	0.983 (0.001)
A2B	Intensity	CTV	0.38 (0.03)	0.085 (0.007)	0.928 (0.006)
		Bladder	2.11 (0.24)	0.47 (0.07)	0.77 (0.03)
		Rectum	0.88 (0.08)	0.118 (0.008)	0.886 (0.007)
	CS	CTV	0.193 (0.022)	0.015 (0.002)	0.985 (0.002)
		Bladder	1.21 (0.43)	0.27 (0.12)	0.85 (0.06)
		Rectum	0.34 (0.07)	0.024 (0.006)	0.974 (0.005)
	CS + P	CTV	0.20 (0.02)	0.012 (0.001)	0.988 (0.001)
		Bladder	0.272 (0.025)	0.022 (0.003)	0.987 (0.001)
		Rectum	0.29 (0.03)	0.018 (0.002)	0.979 (0.002)

*Note*: Values in parentheses are one standard deviation (1 σ).

Abbreviations: CS, controlling structure; CS + P, controlling structure with points; DIR, deformable image registration; DSC, Dice similarity coefficient; DTA_max_, max distance to agreement; DTA_mean_, mean distance to agreement.

**FIGURE 5:**
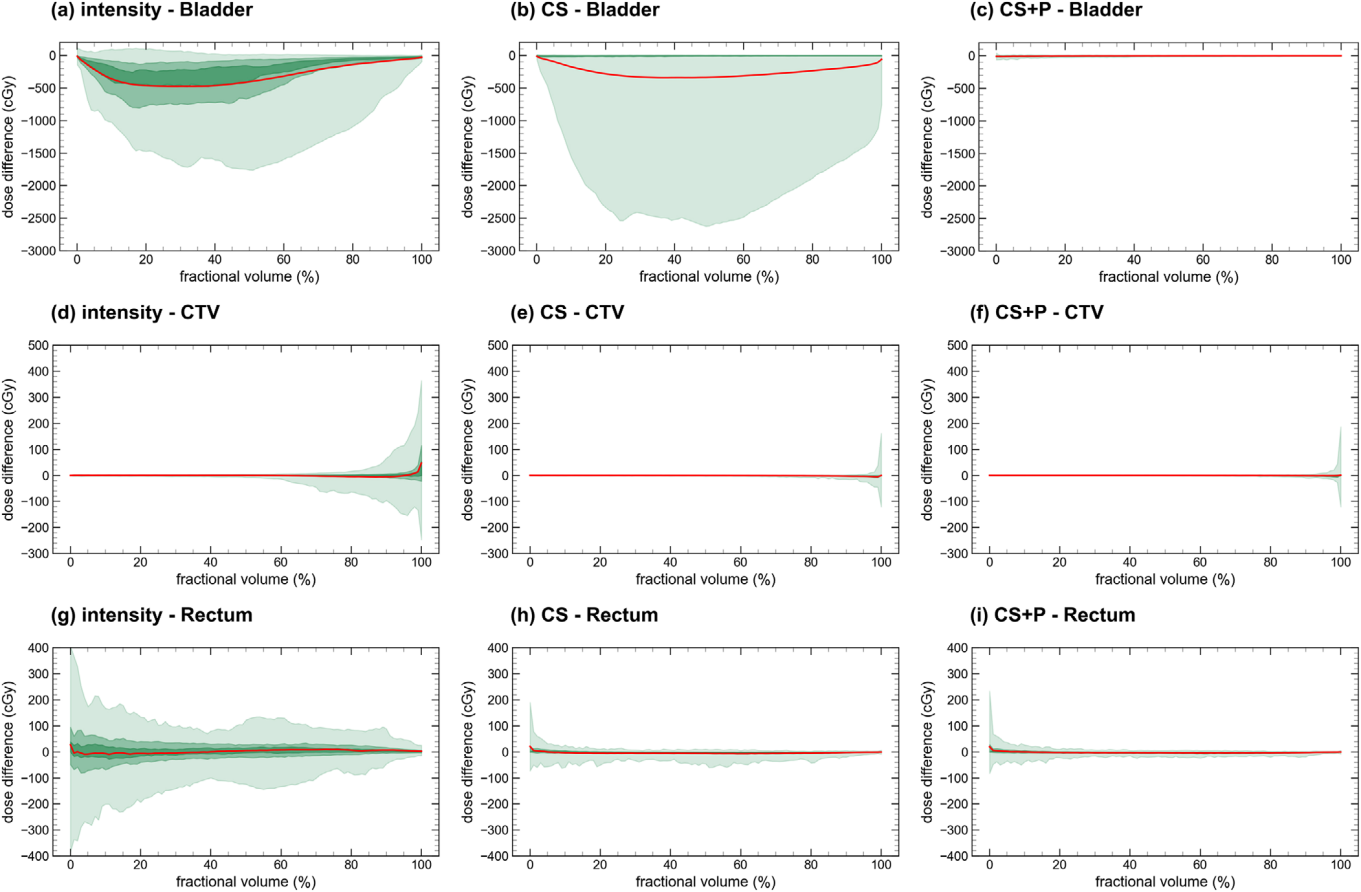
Adapt to beam‐on (A2B) ΔDVH comparison for the three DIR strategies and contours. The green bands provide the 95% (lightest), 50%, and 25% (darkest) confidence intervals, and the bold central red line represents the mean ΔDVH curve. DIR, deformable image registration.

In Figure 5, we present the A2B ΔDVH curves for the bladder, CTV, and rectum for the three DIR strategies (the A2V plots are available in S1). We report the DVH and per‐fraction difference DVH metrics for all image pairs, DIR strategies, and contours in Table S4. We observed improvement of the ΔDVH curves with increasing DIR complexity. Specifically for the A2B mapping, the 95% confidence interval (CI) of the CTV D98% was [−121 cGy, 192 cGy], [−44 cGy, 14 cGy], and [−17 cGy, 21 cGy], bladder D5cc was [−195 cGy, 121 cGy], [−38 cGy, 199 cGy], and [−73 cGy, 12 cGy], and of the rectum D1cc was [−350 cGy, 250 cGy], [−30 cGy, 70 cGy], and [−45 cGy, 56 cGy], reported for intensity‐only, CS, and CS + P. Consistent with the geometry metrics, the CS approach showed a subset of patients with large differences in bladder dose volume metrics (particularly between 20% and 60% of the bladder volume) due to poor bladder contour propagation. CTV ΔDVH variation above 90% relative volume was reduced most between intensity and the CS strategy, demonstrated by the improvement of the 95% CI curve in Figure 5. Similarly, the rectum ΔDVH is most improved when using CS, compared to intensity only.

### 3.2 Inter‐fractional

**FIGURE 6:**
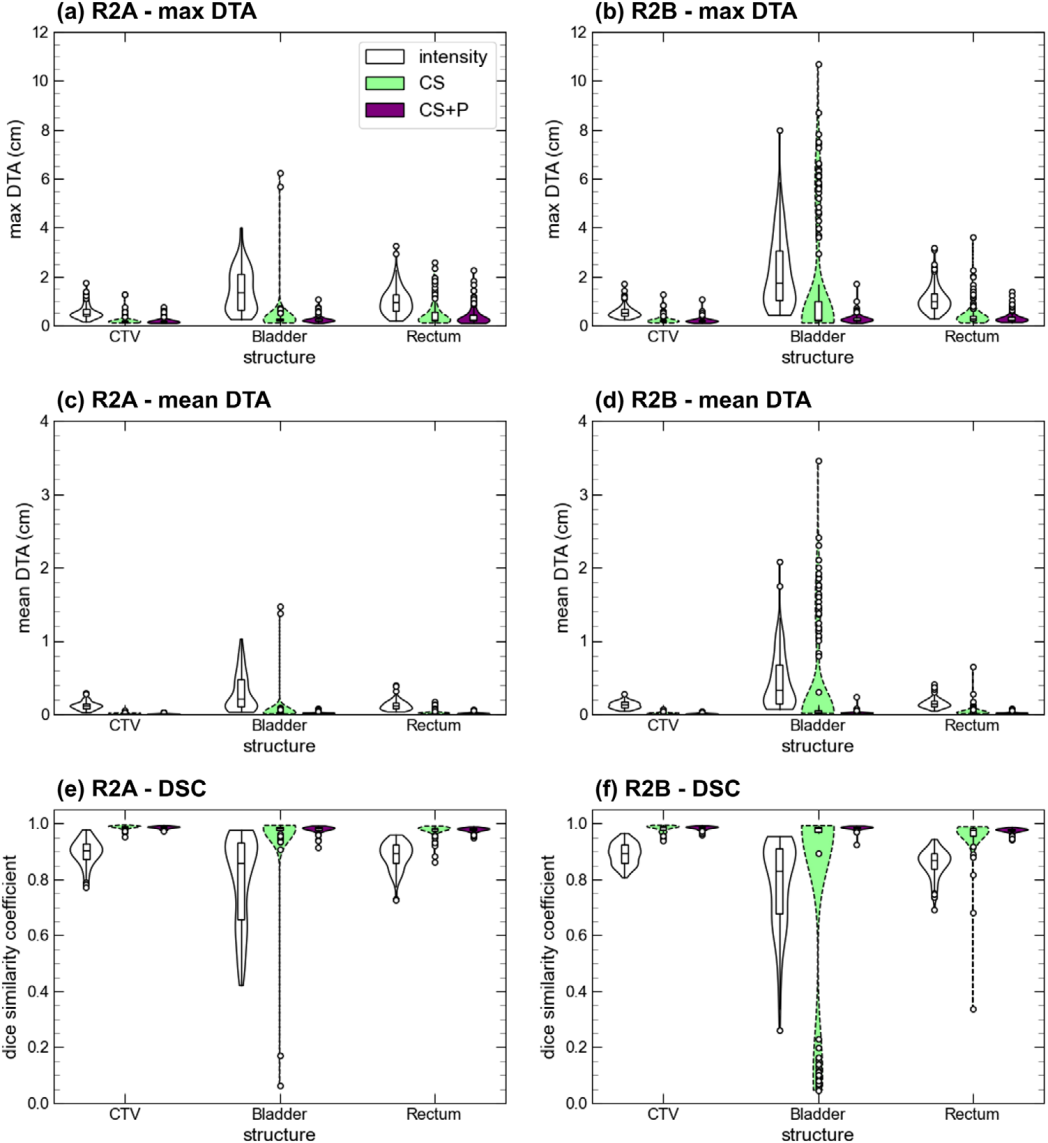
Inter‐fractional geometry comparison for the R2A and R2B image pairs using the three DIR strategies for the CTV, bladder, and rectum. DIR, deformable image registration.

In Figure 6, we present the geometry metrics for the R2A and R2B image pairs for all DIR strategies. Table 2 provides the metric means for the R2A, R2V, and R2B mappings. Similar to the intra‐fractional results, we observed an improvement when using CS for the CTV and rectum. Comparing the intensity to CS DIRs, The DTA_mean_ for the R2B mapping changed from 0.131 ± 0.009 to 0.018 ± 0.002 cm for the CTV and 0.154 ± 0.013 to 0.036 ± 0.013 cm for the rectum. However, for the bladder, though generally improved with the CS approach (DTA_mean_ reducing from 0.46 ± 0.08 to 0.388 ± 0.14 cm for the R2B pair), a subset of fractions performed poorly with outliers most pronounced for the R2B pairing. In Figures S4 and S5, we present the DTA_max_ as a function of relative bladder change for the A2B and R2B mappings for the three DIR strategies. For the intensity‐only DIRs, we saw a consistent increase in DTA_max_ as a function of bladder volume change. For the CS strategy, save for a few fractions, limited DTA_max_ variation existed for <100% bladder volume increase from reference, whereas for >100% bladder volume increase (volume doubling), occurrence of DTA_max_ > 2 cm rose sharply. Applying the CS + P strategy corrected for these bladder deformation errors and reduced the magnitude of the DTA_max_ as a function of bladder volume change (Figure S5c) as well as reduced DTA_mean_ to 0.015 ± 0.001 cm for the CTV, 0.025 ± 0.004 cm for the bladder, and 0.021 ± 0.002 cm for the rectum.

**TABLE 2:** Inter‐fractional geometry comparison metrics of manual and deformed structures for all inter‐fraction image combinations and DIR strategies.

Image pair	DIR	OAR	DTA_max_ (cm)	DTA_mean_ (cm)	DSC
R2A	Intensity	CTV	0.57 (0.06)	0.120 (0.010)	0.899 (0.009)
		Bladder	1.48 (0.18)	0.31 (0.05)	0.80 (0.03)
		Rectum	1.02 (0.11)	0.126 (0.013)	0.886 (0.010)
	CS	CTV	0.19 (0.03)	0.012 (0.001)	0.987 (0.001)
		Bladder	0.34 (0.15)	0.04 (0.04)	0.967 (0.022)
		Rectum	0.45 (0.10)	0.022 (0.005)	0.976 (0.004)
	CS + P	CTV	0.185 (0.021)	0.012 (0.001)	0.987 (0.001)
		Bladder	0.259 (0.027)	0.020 (0.002)	0.981 (0.002)
		Rectum	0.39 (0.07)	0.019 (0.002)	0.978 (0.002)
R2V	Intensity	CTV	0.63 (0.06)	0.143 (0.012)	0.882 (0.010)
		Bladder	1.28 (0.17)	0.23 (0.04)	0.868 (0.021)
		Rectum	0.99 (0.09)	0.149 (0.012)	0.863 (0.010)
	CS	CTV	0.20 (0.03)	0.014 (0.002)	0.985 (0.002)
		Bladder	0.64 (0.29)	0.12 (0.07)	0.93 (0.04)
		Rectum	0.37 (0.07)	0.024 (0.006)	0.972 (0.007)
	CS + P	CTV	0.205 (0.027)	0.013 (0.001)	0.986 (0.001)
		Bladder	0.272 (0.024)	0.022 (0.002)	0.985 (0.001)
		Rectum	0.31 (0.04)	0.018 (0.002)	0.979 (0.001)
R2B	Intensity	CTV	0.57 (0.05)	0.131 (0.009)	0.892 (0.008)
		Bladder	2.21 (0.29)	0.46 (0.08)	0.78 (0.03)
		Rectum	1.10 (0.11)	0.154 (0.013)	0.859 (0.010)
	CS	CTV	0.22 (0.03)	0.018 (0.002)	0.982 (0.002)
		Bladder	1.6 (0.5)	0.388 (0.14)	0.78 (0.08)
		Rectum	0.44 (0.10)	0.036 (0.013)	0.961 (0.013)
	CS + P	CTV	0.222 (0.025)	0.015 (0.001)	0.985 (0.001)
		Bladder	0.29 (0.04)	0.025 (0.004)	0.986 (0.001)
		Rectum	0.33 (0.05)	0.021 (0.002)	0.976 (0.002)

*Note*: Values in parentheses are one standard deviation (1 *σ*).

Abbreviations: CS, controlling structure; CS + P, controlling structure with points; DIR, deformable image registration; DSC, Dice similarity coefficient; DTA_max_, max distance to agreement; DTA_mean_, mean distance to agreement.

**FIGURE 7:**
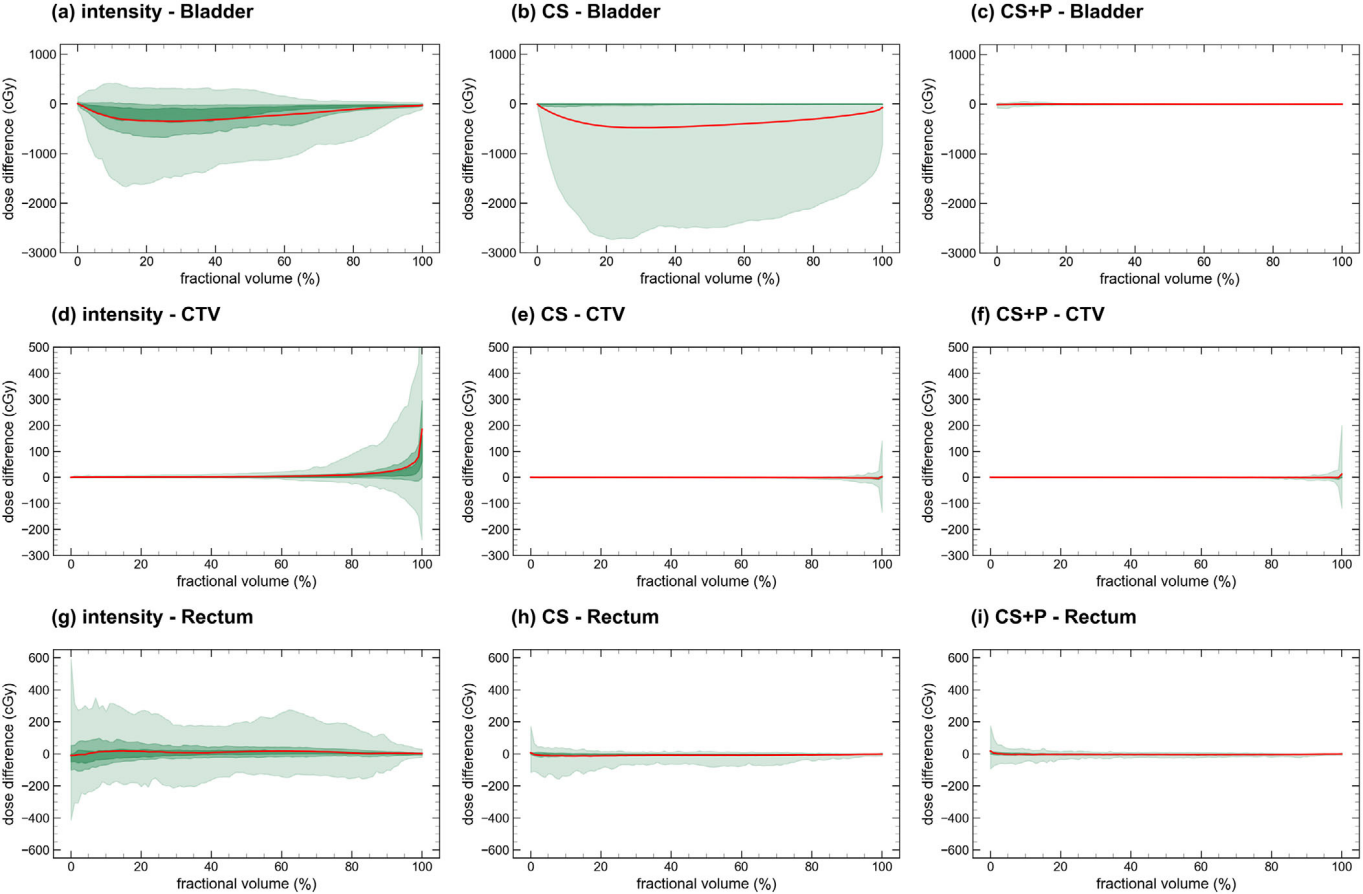
Reference to beam‐on (R2B) ΔDVH comparison for the three DIR strategies. The green bands provide the 95% (lightest), 50%, and 25% (darkest) confidence intervals, and the bold central red line represents the mean ΔDVH curve. DIR, deformable image registration.

In Figure 7, we present the R2B ΔDVH curves for the bladder, CTV, and rectum for the three DIR strategies (R2A and R2V plots are detailed in Figures S2 and S3). The DVH and per‐fraction difference DVH metrics for all image pairs, DIR strategies, and contours are reported in Table S5. The ΔDVH curves showed improvement with increasing DIR complexity, consistent with the geometry metrics. Specifically, the 95% CI intensity‐only, CS, and CS + P for the CTV D98% were [−126 cGy, 389 cGy], [−29 cGy, 19 cGy], and [−18 cGy, 26 cGy], for bladder D5cc were [−190 cGy, 245 cGy], [−51 cGy, 544 cGy], and [−79 cGy, 36 cGy], and for rectum D1cc were [−319 cGy, 247 cGy], [−106 cGy, 72 cGy], and [−52 cGy, 74 cGy]. Intensity‐only distributions for the CTV and rectum were broader for the CTV and rectum compared to the intra‐fraction results. Both CS and CS + P strategies produced comparable results to the intra‐fractional data across all structures.

## 4 DISCUSSION

In this study, we performed a comprehensive analysis of multiple DIR strategies, including a unique hybrid structure and point DIR, applied to a cohort of patients with prostate cancer receiving MR‐guided adaptive radiotherapy. Our overarching goal was to demonstrate a general methodology for geometric and dosimetric evaluation of DIR accuracy and to arrive at a validated DIR strategy for use in future dose accumulation studies in this patient cohort. A key strength of this work is the inclusion of multiple intra‐ and inter‐fraction DIRs across 125 fractions with three different strategies for 1875 DIRs in total. Applying the DVFs to CTV, bladder, and rectum enabled comparison against expert manual contours using geometry and DVH metrics. Comparing DIR strategies, our novel controlling structure and point hybrid DIR strategy offered optimal DIR performance for both intra‐ and inter‐fraction comparison.

In the context of clinical dose accumulation, inclusion of structures or points in the DIR must be justified based on the potential resource burden of segmenting key structures on verification or beam‐on images. Our results show that intensity‐only DIR produced average DTA_mean_ values < 1.5 mm and DSC scores > 0.859 for the CTV and rectum structures for all image pairs. The bladder structure produced mean DTA_mean_ values below 3.1 mm and DSC scores above 0.80 for the A2V, R2A, and R2V mappings, while the mappings to the MR_beam‐on_ resulted in worse geometry metrics. AAPM task group 132 provides guidance on DIR evaluation and indicates a tolerance for DTA_mean_ in the range of 2–3 mm and for DSC values above 0.8–0.9.^11^ Our intensity‐only DIRs for the CTV and rectum would meet these geometric criteria. However, when we incorporate DVH analysis for the intensity‐only DIRs, we find that the different 95% CI for CTV D98% was as broad as [−126 cGy, 389 cGy], rectum D1cc was [−190 cGy, 245 cGy] and bladder D5cc was [−318 cGy, 247 cGy]. For the CTV, this D98% variation translates to a difference of −4.2 to 13.0% of the 3000 cGy prescribed dose for our cohort. This demonstrates the importance of placing DIR validation in context of the delivered dose distribution and inspecting the entire population geometry metric distribution, as mean values may obscure individual patient/fraction deviations.^19^, ^20^ Comparatively, Christiansen et al. looked at conventionally fractionated prostate radiotherapy, obtaining MR scans throughout their treatment course and, using the default Monaco DIR algorithm, obtained DSC values of 0.9, 0.87, and 0.92 and DTA_mean_ values of 1.0, 1.25, and 1.11 mm for the prostate, rectum, and bladder, respectively, when comparing deformed and manual contours.^21^ Additionally, Saito et al. generated adapted plans on the MR_adapt_ using only the deformed structures from the MR_reference,_ produced by the Monaco TPS, and subsequently evaluated the resulting plan using manual contours on the same image.^22^ They obtained DSC values of 0.92–0.99, 0.75–1.0, and 0.31–1.0 for the prostate, rectum, and bladder. Further, they observed that the near max bladder and rectum dose metrics failed on roughly 6% of cases when evaluated on the manual contours. Collectively, these geometric results for the rectum and prostate are in line with our study. The bladder potentially improved compared to our intensity‐only DIRs; however, these studies did not include a dosimetric evaluation to compare to our work. Previous reports have highlighted importance of DIR strategy evaluation beyond geometric analysis, including DVH analysis and visual inspection, as key DIR validation elements.^9^, ^23^, ^24^ Murr et al. multi‐institution comparison DSC values for the two prostate cases were 0.89–0.98 for CTV, 0.69–0.98 for rectum and 0.7–0.99 for bladder, with the largest dosimetric differences observed for bladder V28Gy of 10.2% for prostate case 1 and 7.6% for prostate case 2.

To improve the DIR accuracy, we used the CTV, bladder, and rectum as input for the CS DIR strategy. We found this resulted in improved contour deformation for the CTV and rectum (mean DTAmean < 0.36 mm and DSC > 0.961 for all image pairs). The use of controlling structures has been shown to improve DIR mapping in abdominal and pelvic sites.^25^, ^26^ Bohoudi et al. showed that CS DIR strategy for MR‐to‐MR mapping improved dose accumulation results for the rectum and bladder^19^ using film measurements on an anthropomorphic phantom. However, in our cohort, although there is a general improvement in the propagated bladder structures, a subset of fractions had substantial contour deviations resulting in a mean DTAmean as large as 3.9 mm and DSC as low as 0.78. Overall, we found that DIR performance was the worst for registrations with MR_beam‐on_, in which bladder volume differences between fixed and moving images were greatest, suggesting that bladder volume change is contributing to poor DIR performance for certain fractions. We inspected the relationship between the DTA_max_ and relative bladder volume change and observed for the intensity‐only DIRs that DTA_max_ consistently increased with increasing change in bladder volume (Figure S4). For the CS strategy, a sharp decrease in DIR performance is observed when the bladder doubles in size across image pairs. However, many DIRs performed well for the large bladder deformations beyond this threshold. This suggests that there is a component of patient‐specific bladder anatomy impacting the CS DIR performance. Adaptive prostate treatment sessions can exceed 50 min, over which continued bladder filling occurs.^27^ Patients in our study received ART‐specific bladder preparation instructions, and with rigorous instruction and management, instances of restarting adaptive sessions reduced but did not significantly change the initial bladder volumes.^28^ The impact of more stringent bladder filling may translate into improved CS DIR performance, but as noted above, the bladder shape may be the substantive contributing factor to poor DIRs.

The RayStation ANACONDA algorithm uses a chamfer matching distance metric for the CS algorithm, which has been observed to produce erroneous DIRs in the case of large deformations.^29^ Starting with the RayStation 2023B version, an additional image similarity metric has been added to help mitigate this issue and would require further validation for this application.^12^ In the absence of this updated algorithm, we propose a controlling point generation algorithm to produce guiding points on the surfaces of the rectum, CTV, and bladder structures to drive the CS + P DIR strategy. We show this DIR approach corrects for the bladder deformation errors, DSC above 0.97 and DTA_mean_ below 0.21 mm for all image pairs, while maintaining performance for the prostate and rectum. Comparing the R2B CS and CS + P DIRs, we see a change in the 95% CI of the CTV D98% from [−29 cGy, 19 cGy] to [−18 cGy, 26 cGy], rectum D1cc from [−106 cGy, 72 cGy] to [−52 cGy, 74 cGy], and bladder D5cc from [−51 cGy, 544 cGy] to [−79 cGy, 36 cGy]. The D98% results are within 1%–2% of the 3000 cGy clinical prescription when using the CS + P approach. The change in the mean difference of the metrics (Table S5) for all structures between the CS and CS + P is within 40 cGy. However, the reduction in the 95% CI bladder D5cc between CS and CS + P is over 500 cGy, highlighting the benefit of the CS + P by providing enforcement of corresponding points across the surface of the bladder. The introduction of point to the CS DIR helps overcome the chamfer matching objective function, informed by the distance maps between the contours on the two images, from pushing the deformed surface toward the inferior wall of the bladder.^12^ Based on the improved geometry and DVH analysis of the CS + P DIR, we selected this strategy as the approach for future dose accumulation work on this cohort.

Though the CS + P strategy is generalizable to other sites and image modality pairs, DIR evaluation per site, dose distribution, and relevant clinical DVH metrics would be necessary. Not all disease sites will be impacted by systematic organ changes over the adaptive treatment course, as in pelvic sites, and may face other challenges such as the influence of peristaltic motion. For liver and upper gastrointestinal targets, intensity‐only or CS approaches may yield acceptable results when confined to an area of interest for DVH analysis.^26^, ^30^ Incorporation of controlling points or use of the updated ANACONDA algorithm for these sites could be of interest for future exploration.

Controlling structure approaches hinge on the availability of contours across paired images to drive the controlling point generation and the DIR mapping. This introduces observer contouring variability and currently limits automation of the CS + P DIR strategy. For expert contour delineation, MR‐based inter‐observer variability was evaluated by Pathmanathan et al.^31^ for prostate with a DSC of 0.94 and DTA_max_ of 4.8 mm, and Sanders et al.^32^ for the prostate, rectum, and bladder with DSC of 0.904, 0.893, and 0.928, respectively. Christiansen et al. evaluated MR‐based intra‐observer variability with DSC of 0.92, 0.95, and 0.97, DTA_mean_ of 0.88, 0.65, and 0.55 mm, and DTA_max_ of 4.89, 7.65, and 4.05 mm for the prostate, rectum, and bladder.^21^

Automated contouring for both conventional and adaptive treatment planning is seeing a substantial uptake in the field. As McCulloch et al. proposed for liver patients, we expect that using auto‐contours to establish structures for the CS/CS + P approaches will reduce or eliminate manual intervention.^26^ Though requiring further validation, if using auto contouring specifically to drive DIR mapping, we hypothesize that even if differences between automated and manual contours exist, so long as the automated contours are anatomically consistent for a given patient across fractions and within‐session images, the resulting CS/CS + P DIRs would likely be acceptable. The use of sufficiently reliable automated contouring would address the limitations of the CS/CS + P approach and allow the process to be run passively on a per‐patient basis with minimal user inspection or intervention. Importantly, timing considerations suggest that such integration is clinically feasible: while intensity‐only DIR requires 9 s per image pair, the CS approach increases this to 13 s, and the CS + P strategy remains comparable at 10 s, even when accounting for additional processing (e.g., mesh generation: 6 s per structure, point generation: 5 s per structure). As summarized in Table S7, the time increase relative to intensity‐only DIR is modest. The main barriers to routine clinical implementation of the CS + P strategy are the need for images and doses to be translated into the RayStation TPS, contours to be generated across all images, and generation of points across these surfaces. As noted, contour generation efforts may be addressed via auto contouring implementation. Data transfer, point generation, and subsequent DIR creation may be automated through additional scripts and tools and are well within the practical constraints of online adaptive workflows, making CS/CS + P strategies potentially attractive for clinical adoption. Extending this DIR strategy to register regions of interest other than the prostate CTV, rectum, and bladder might be possible. Any controlling ROI strategy, with or without points, requires anatomically consistent contours on all registered images. In addition, whole‐organ registration, which would be necessary for accurate accumulation of relative‐volume DVH metrics, requires capturing the entire structure within the imaging field‐of‐view to allow for complete segmentation. This may be challenging for luminal structures, such as the sigmoid or small bowel, due to a lack of well‐defined anatomical landmarks and complex inter‐fraction motion. For example, the entire small bowel may not be captured within the field of view, and substantial rearrangement of bowel loops might increase the difficulty of identifying the same segment of bowel between images that span many days. Furthermore, the rearrangement of bowel loops might not be possible to accurately model using a smooth deformation vector field that is only designed to represent local expansion and compression. Bowel structures were not routinely contoured in our clinical dataset, limiting our ability to study whether the CS + P method provides an advantage for registering these structures. Developing robust registration strategies for luminal organs remains an important area for future work.

With this study, we have demonstrated an approach to estimate the dosimetric uncertainty of DIR for the application of dose accumulation by comparing boundary alignment and dose–volume histogram differences between DIR‐mapped contours and reference manual segmentations. A complete assessment of DIR accuracy for dose accumulation and 3D dose warping within an anatomical structure generally requires a 3D evaluation of both surface alignment and volumetric alignment throughout the region of interest.^33^ A limitation of our study is that we did not perform a quantitative assessment of volumetric alignment throughout the ROIs.

The volumetric accuracy of MR‐MR prostate DIR using the RayStation hybrid DIR algorithm used in our evaluation was previously reported.^34^ The use of controlling ROIs yielded a DTA_mean_ of 0.4 mm at the prostate boundary and mean target registration error (TRE) of 3.9 mm at fiducial markers within the prostate interior. The corresponding dosimetric uncertainty of deformable dose warping within the prostate was not evaluated. The MR images registered in that study were acquired with and without an endorectal MR coil. The impact of the endorectal coil may have resulted in larger anatomical differences between the image pairs as compared to our study, where consistent imaging setups and no endorectal coil were used for all images. In another study evaluating volumetric DIR error using the RayStation hybrid DIR algorithm for CT‐CBCT registration using consistent imaging setups, a mean TRE of 2 mm within the prostate was reported.^35^ In a multi‐institutional study evaluating volumetric DIR error for MR‐MR prostate registration using six DIR algorithms (different than the RayStation hybrid DIR algorithm), mean TRE at fiducial markers within the prostate ranged from 2 to 7 mm.^36^

The extent to which spatial DIR error translates to dosimetric error depends on the magnitude of local dose gradients in the dose distribution to be warped. Treatment plans for ultra hypo‐fractionated prostate radiotherapy typically yield uniform dose distributions within the prostate PTV (clinical goals of D95 > 100% and D2 < 112% in this study) with maximal dose fall‐off beyond the PTV boundary for OAR sparing. As such, we assume the dosimetric sensitivity of prostate DIR error is dominated by spatial errors near the high dose gradients at the prostate boundary and insensitive to millimeter‐scale spatial misalignment in relatively uniform dose regions such as the prostate interior.

In this study, we also estimate dosimetric uncertainty of DIR within rectum and bladder ROIs. These organs are “wall structures” where the physiological organ tissue is located within a few millimeters of the organ boundary. For such structures, we assume that the dose interior to the organ wall (e.g., dose to urine or fecal content) is not clinically relevant and that evaluation of DIR accuracy and associated dosimetric uncertainty is most important near the organ boundaries.

The radiobiological importance of volumetric DIR alignment is highly specific to each body region, treatment technique, and organ for which dose is warped and accumulated. Treatment sites and organs other than those studied in this work may warrant additional evaluation of volumetric DIR accuracy, such as the inclusion of manually defined point‐of‐interest landmarks through the ROI volume in addition to manual boundary segmentations.

## 5 CONCLUSIONS

In this study, we have rigorously analyzed DIR strategies for MR‐guided radiotherapy prostate patients, focusing on the evaluation of intensity‐only, controlling structure (CS), and controlling structure with points (CS + P). We demonstrated improved performance of the CS + P strategy, which better accounts for bladder filling during the adaptive fraction. We incorporate and highlight the importance of including dose metrics in DIR validation, where use of only geometry metrics may obscure DIR inaccuracies. Our dosimetric analysis shows a 1%–2% uncertainty (95% CI) in clinically relevant dose metrics for the CS + P DIR strategy. The selected methodology can be used for future dose accumulation studies of MRL prostate patients, and applicability may be expanded by introduction of auto contouring.

## AUTHOR CONTRIBUTIONS

Victor N. Malkov, Iymad R. Mansour, Peter Chung, Jeff D. Winter, and Tony Tadic contributed through project conceptualization, organization, data processing and collection, and manuscript creation and review. Vickie Kong, Winnie Li, Jennifer Dang, Parisa Sadeghi, Inmaculada Navarro, and Jerusha Padayachee contributed to contouring, data curation and collection, and manuscript review.

## CONFLICT OF INTEREST STATEMENT

The authors declare no conflicts of interest.

## Supporting information



Supporting Information
