# Supplementary material for: Evaluation of hybrid DIR performance using controlling structures and points of interest in MR‐guided adaptive radiotherapy for prostate cancer patients
Source: J Appl Clin Med Phys. 2026 Jan 14;27(1):e70437. doi: 10.1002/acm2.70437 (PMC12802555; doi:10.1002/acm2.70437)
Supplement: Supplementary file 1 — Supporting Information [file ACM2-27-e70437-s001.docx]

# Supplemental Material

Table S1: Patient demographics

| **Measure** | **Value** |
| --- | --- |
| Mean Age (± STD) | 70 ± 5 years |
| T-Stage (cases) | T1c (12), T2 (12), T3a (1) |
| N-Stage (cases) | N0 (25) |
| Gleason (cases) | 6 (1), 7 (23), 8 (1) |
| Prostate volume (± STD) | 46 ± 22 mL |
|  |  |
| Mean MR Sim bladder volume (± STD) | 124 (67-80) mL |

Table S2: Institutional clinical goals for 3000 cGy in 5 fractions prostate SBRT

| **Structure** | **Metric** | **Criteria** |
| --- | --- | --- |
| CTVp_3000 | D95 | > 3300cGy |
| PTVp_3000 | D0cc | < 4000cGy |
|  | D2 | < 3500cGy |
|  | D95 | > 3000cGy |
|  | D98 | > 2850cGy |
| Rectum | D50 | < 1000cGy |
|  | D20 | < 2000 cGy |
|  | D1cc | < 3000 cGy |
| Bladder | D40 | < 1500 cGy |
|  | D5cc | < 3000 cGy |
| Femur_L/R | D5 | < 1200 cGy |
| SmallBowel | D1cc | < 2500 cGy |
| LargeBowel | D1cc | < 2500 cGy |
| PenileBulb | D50 | < 2400 cGy |
| PenileBulb | D1cc | < 3000 cGy |
| Urethra | D50 | < 3500 cGy |

Table S3: MR Sequence parameters

| **Parameter** | **T_2_-weighted 6-minute scan** | **T_2_-weighted 2-minute scan** |
| --- | --- | --- |
| Field-of-view (mm) | 400 x 400 x 250 | 400 x 400 x 300 |
| Echo time (ms) | 82 | 278 |
| Repetition time (ms) | 1300 | 1535 |
| SENSE | 4 (phase), 1.2 (slice) | 3.6 (phase) |
| Half-scan | 0.6 | 0.625 |
| Acquisition resolution (mm) | 1.2 x 1.2x1.2 | 1.2 x 1.2x1.2 |
| Reconstructed resolution (mm) | 0.5 x 0.5 x 0.6 | 0.5 x 0.5 x 1.0 |
| Acquisition time | 6 min 7 s | 1 min 57 s |


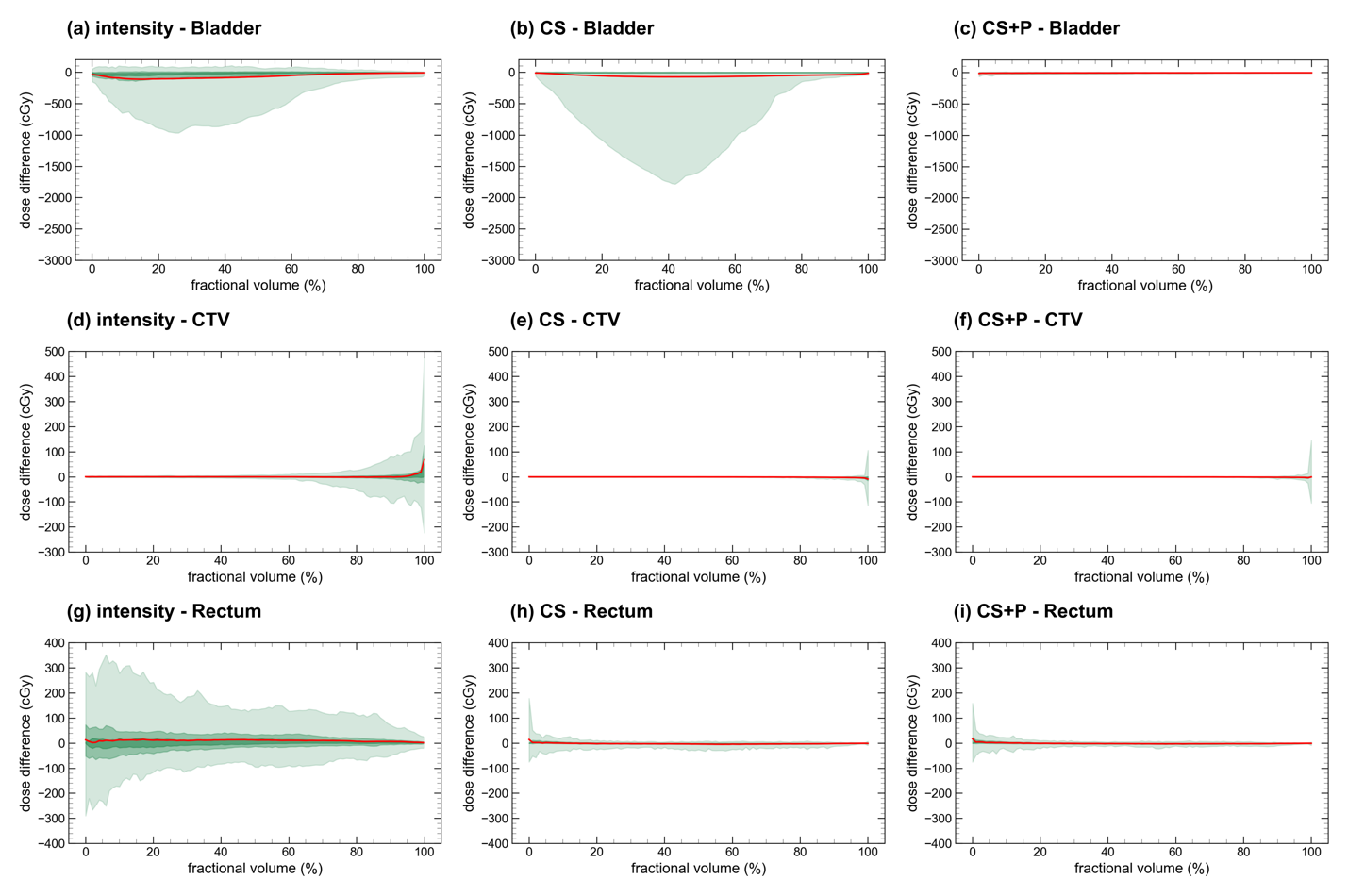


Figure S1: adapt to verify (A2V) ΔDVH comparison for the three DIR strategies. The green bands provide the 95% (lightest), 50% and 25% (darkest) confidence intervals, and the bold central red line represents the mean ΔDVH curve.

Table S4: DVH metrics and per fraction DVH metric difference means for intra-fraction image combinations for the intensity, CS, CS+P DIR strategies. Values in parentheses are one standard deviation (1 σ).

| **image pair** | **DIR** | **OAR** | **Metric** | **Manual mean (cGy)** | **Mapped mean (cGy)** | **Mean Manual – Mapped (cGy)** |
| --- | --- | --- | --- | --- | --- | --- |
| A2V | intensity | CTV | D98% | 3150 (270) | 3140 (280) | 10 (110) |
|  |  | Bladder | D5cc | 2990 (230) | 3030 (220) | -43 (93) |
|  |  | Rectum | D1cc | 2240 (660) | 2250 (650) | -0 (140) |
|  | CS | CTV | D98% | 3150 (270) | 3160 (270) | -3.7 (6.3) |
|  |  | Bladder | D5cc | 2990 (230) | 2990 (240) | 1 (41) |
|  |  | Rectum | D1cc | 2240 (660) | 2240 (670) | 7 (17) |
|  | CS+P | CTV | D98% | 3150 (270) | 3150 (270) | -2.2 (7.0) |
|  |  | Bladder | D5cc | 2990 (230) | 2990 (230) | -6 (12) |
|  |  | Rectum | D1cc | 2240 (660) | 2230 (670) | 7 (18) |
| A2B | intensity | CTV | D98% | 3120 (280) | 3120 (270) | 8 (75) |
|  |  | Bladder | D5cc | 3010 (270) | 3030 (270) | -21 (88) |
|  |  | Rectum | D1cc | 2220 (650) | 2220 (650) | 0.0 (150) |
|  | CS | CTV | D98% | 3120 (280) | 3130 (280) | -5.0 (14) |
|  |  | Bladder | D5cc | 3010 (270) | 3000 (290) | 15 (91) |
|  |  | Rectum | D1cc | 2220 (650) | 2210 (650) | 11 (26) |
|  | CS+P | CTV | D98% | 3120 (280) | 3130 (280) | -1 (11) |
|  |  | Bladder | D5cc | 3010 (270) | 3020 (260) | -8 (22) |
|  |  | Rectum | D1cc | 2220 (650) | 2210 (650) | 9 (27) |


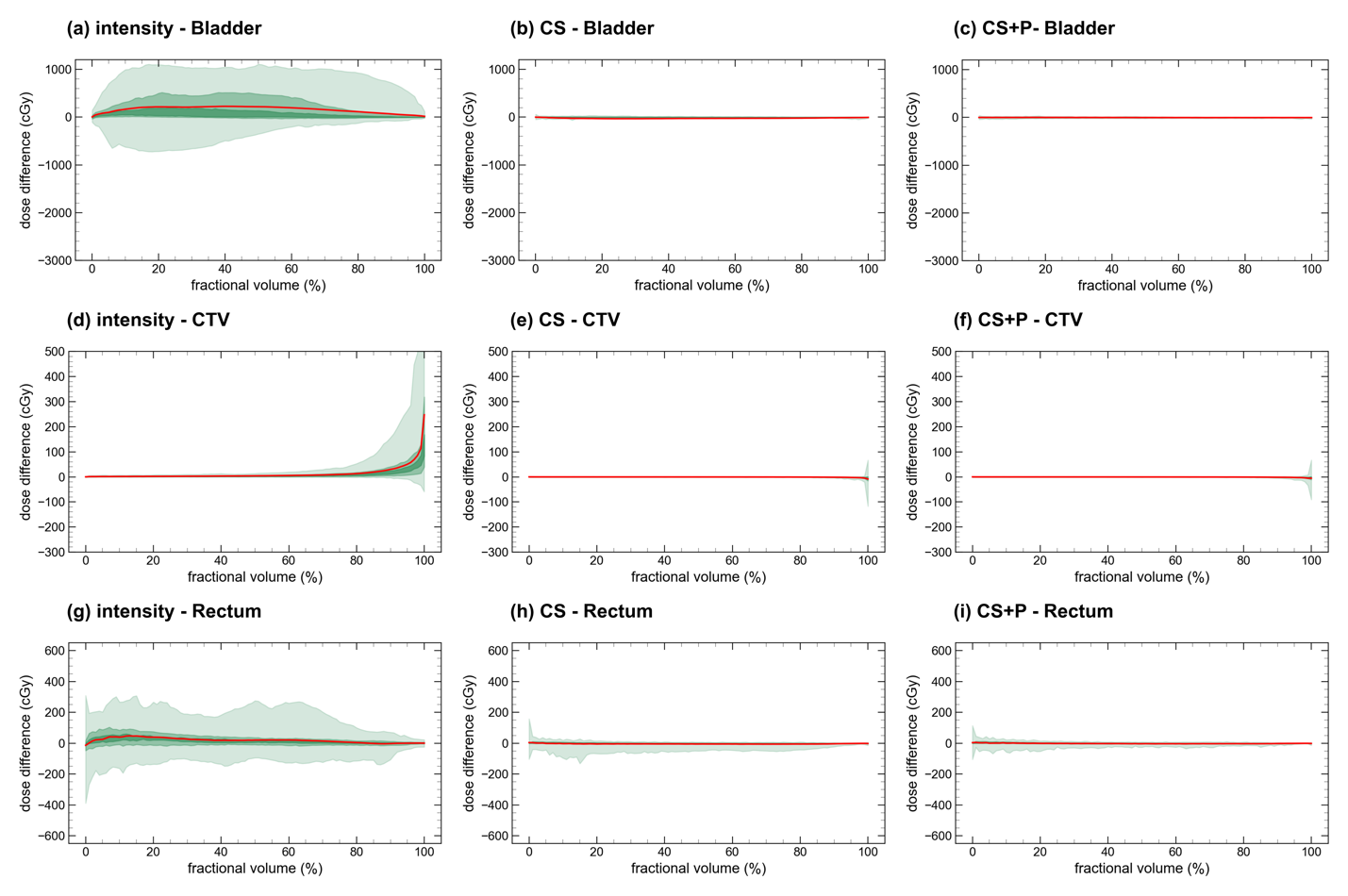


Figure S2: Reference to adapt (R2A) ΔDVH comparison for the three DIR strategies. The green bands provide the 95% (lightest), 50% and 25% (darkest) confidence intervals, and the bold central red line represents the mean ΔDVH curve.


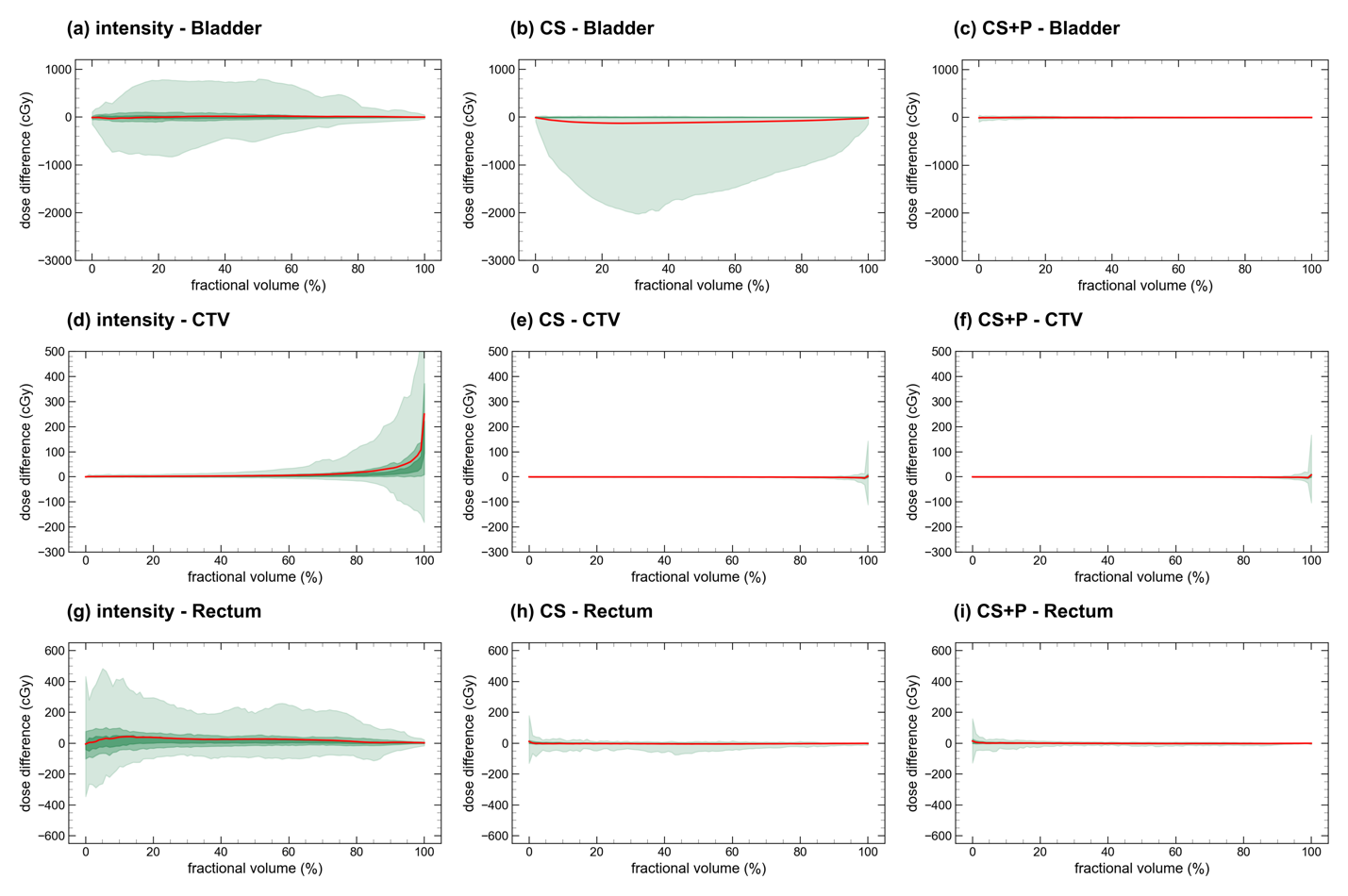


Figure S3: Reference to verify (R2V) ΔDVH comparison for the three DIR strategies. The green bands provide the 95% (lightest), 50% and 25% (darkest) confidence intervals, and the bold central red line represents the mean ΔDVH curve.

Table S5: DVH metrics and per fraction DVH metric difference means for inter-fraction image combinations for the intensity, CS, CS+P DIR strategies. Values in parentheses are one standard deviation (1 σ).

| **image pair** | **DIR** | **OAR** | **Metric** | **Manual mean (cGy)** | **Mapped mean (cGy)** | **Mean Manual – Mapped (cGy)** |
| --- | --- | --- | --- | --- | --- | --- |
| R2A | intensity | CTV | D98% | 3265 (32) | 3180 (150) | 90 (140) |
|  |  | Bladder | D5cc | 2890 (190) | 2850 (240) | 40 (160) |
|  |  | Rectum | D1cc | 2530 (570) | 2540 (560) | -10 (100) |
|  | CS | CTV | D98% | 3265 (32) | 3269 (31) | -3.5 (4.7) |
|  |  | Bladder | D5cc | 2890 (190) | 2880 (190) | 10 (53) |
|  |  | Rectum | D1cc | 2530 (570) | 2520 (570) | 5 (24) |
|  | CS+P | CTV | D98% | 3265 (32) | 3269 (31) | -3.6 (5.8) |
|  |  | Bladder | D5cc | 2890 (190) | 2890 (180) | 2 (16) |
|  |  | Rectum | D1cc | 2530 (570) | 2520 (580) | 5 (24) |
| R2V | intensity | CTV | D98% | 3150 (270) | 3070 (340) | 90 (220) |
|  |  | Bladder | D5cc | 2990 (230) | 3000 (240) | -10 (130) |
|  |  | Rectum | D1cc | 2240 (660) | 2260 (650) | -20 (160) |
|  | CS | CTV | D98% | 3150 (270) | 3160 (270) | -4 (11) |
|  |  | Bladder | D5cc | 2990 (230) | 2970 (260) | 12 (90) |
|  |  | Rectum | D1cc | 2240 (660) | 2240 (660) | 4 (25) |
|  | CS+P | CTV | D98% | 3150 (270) | 3150 (270) | -2.3 (8.0) |
|  |  | Bladder | D5cc | 2990 (230) | 2990 (220) | -6 (25) |
|  |  | Rectum | D1cc | 2240 (660) | 2240 (670) | 6 (21) |
| R2B | intensity | CTV | D98% | 3120 (280) | 3070 (300) | 60 (130) |
|  |  | Bladder | D5cc | 3010 (270) | 3010 (290) | 10 (110) |
|  |  | Rectum | D1cc | 2220 (650) | 2240 (640) | -20 (160) |
|  | CS | CTV | D98% | 3120 (280) | 3130 (280) | -3 (15) |
|  |  | Bladder | D5cc | 3010 (270) | 2970 (310) | 40 (130) |
|  |  | Rectum | D1cc | 2220 (650) | 2220 (650) | -1 (43) |
|  | CS+P | CTV | D98% | 3120 (280) | 3130 (280) | -1 (14) |
|  |  | Bladder | D5cc | 3010 (270) | 3020 (260) | -6 (25) |
|  |  | Rectum | D1cc | 2220 (650) | 2220 (650) | 5 (33) |

Table S6: Deformable image registration timing

| **Item** | **Timing (s)** |
| --- | --- |
| Intensity only DIR per image pair | 9 (reference <–> Adapt) |
| Controlling structure DIR per image pair | 13 (reference <–> Adapt) |
| POI generation per structure | 5 (Run for CTV) |
| Mesh generation | 19 (Bladder, Rectum, CTV for Reference, Adapt, Verify, and Beam On) |
| Controlling structure and points DIR per image pair | 10 (reference to adapt) |


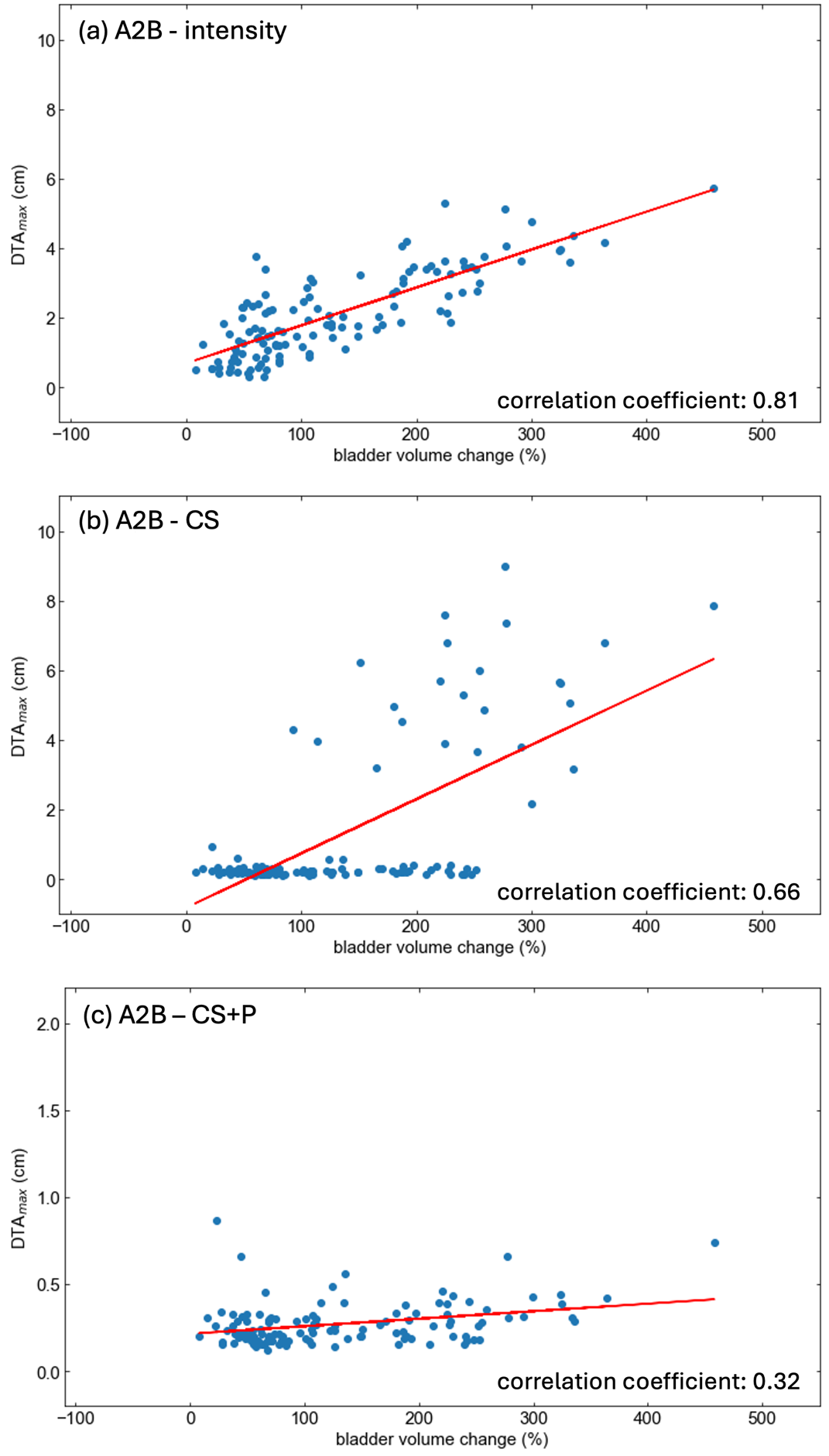


Figure S4: DTA_max_ as a function of bladder volume change between the MR_adapt_ and the MR_beam-on_ for the (a) intensity (b) CS and (c) CS+P DIR strategies. Note the change of scale in (c).


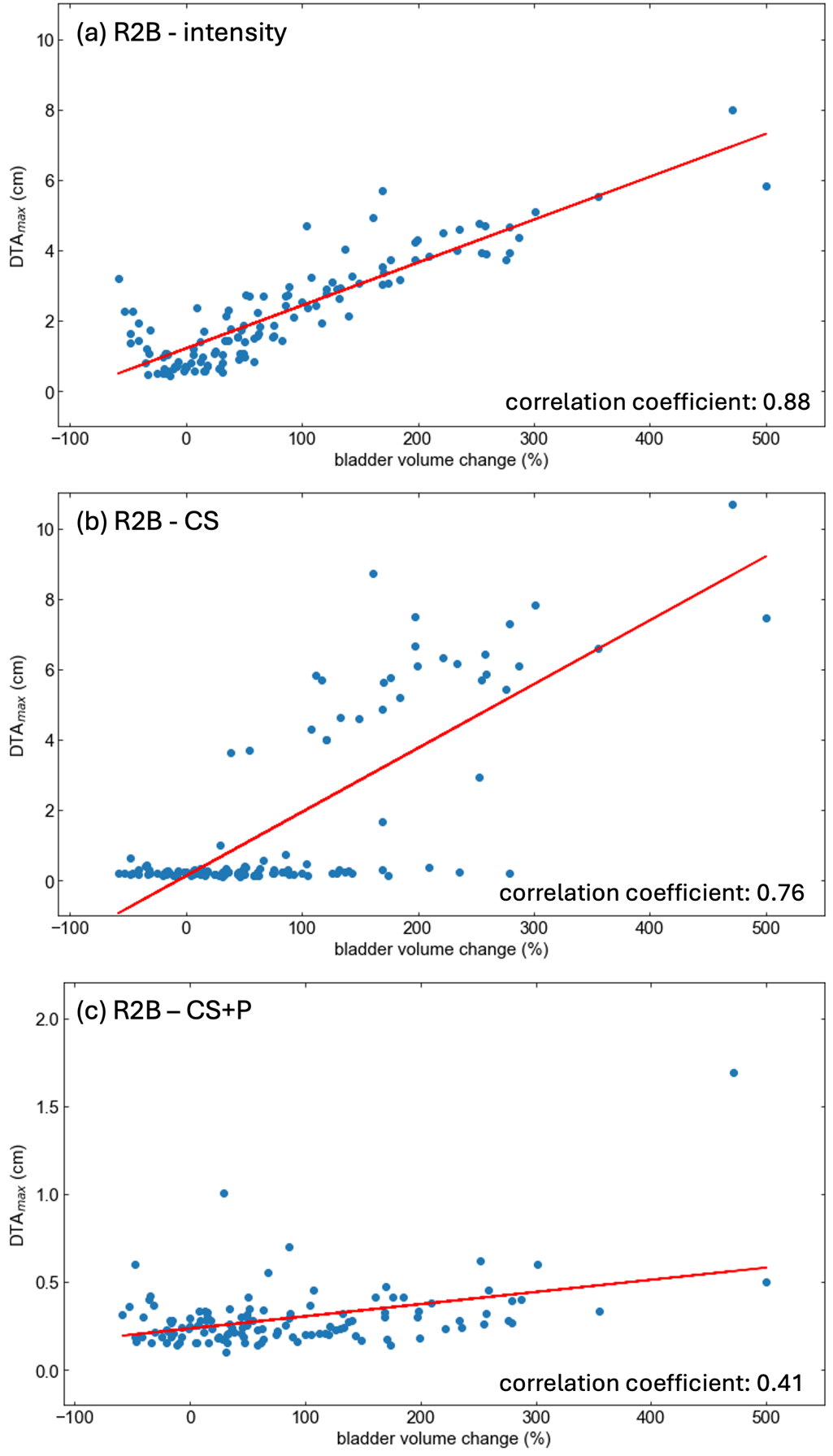


Figure S5: DTA_max_ as a function of bladder volume change between the MR_ref_ and the MR_beam-on_ for the (a) intensity (b) CS and (c) CS+P DIR strategies. Note the change of scale in (c)

**Patient Inclusion Criteria associated with NCT00913939:**

- Prior enrollment of UHN 05-0641-C or UHN 12-5015-C (Arm 1)
- Histological evidence of recurrent prostate adenocarcinoma (Arm 1)
- PSA doubling time > 6 months (Arm 1)
- High-risk localized prostate cancer (>T2 or G>7 or PSA>20) (Arm 2)
- Planned for EBRT + HDR boost (+/- hormone therapy) (Arm 2)
- ECOG 0 or 1
- Age > 18 years
- Informed consent: All patients must sign a document of informed consent indicating their understanding of the investigational nature and risks of the study before any protocol related studies are performed.

**Patient Exclusion Criteria associated with NCT00913939:**

- Radiological evidence of regional or distant metastases
- Contraindications to MRI (Patient weighing >136kg (scanner weight limit), Patients with pacemakers, cerebral aneurysm clips, shrapnel injury, or implantable electronic devices not compatible with MRI)
- Bleeding diathesis and anti-coagulative therapy that cannot be temporarily ceased during brachytherapy
- Previous prostate brachytherapy
- Active hormonal therapy (Arm 1)
- >50% of contiguous sextants involved with tumor (Arm 1)
- Previous pelvic radiotherapy (Arm 2)
- Contraindications to endorectal coil, surgically absent rectum, severe hemorrhoids or colorectal surgery.
- Latex Allergy
- Contraindications to conscious sedation, local anesthesia, or spinal/epidural anesthesia.
- IPSS >18
- Large TURP defect
- TURP within the past 6 months
- Prostate gland size >80cc
- History of Ulcerative Colitis, Crohn's Disease, Ataxia Telangiectasia, or SLE
- Other medical conditions deemed by the PI to make patient ineligible for MRI-guided Prostate HDR brachytherapy.
